# Dandelion Polysaccharides Ameliorate High-Fat-Diet-Induced Atherosclerosis in Mice through Antioxidant and Anti-Inflammatory Capabilities

**DOI:** 10.3390/nu15194120

**Published:** 2023-09-24

**Authors:** Shuaishuai Zhou, Zi Wang, Yanling Hao, Peng An, Junjie Luo, Yongting Luo

**Affiliations:** 1 Department of Nutrition and Health, China Agricultural University, Beijing 100193, China; sszhou_2020@163.com (S.Z.); zxcvbnm7895123hh@163.com (Z.W.); haoyl@cau.edu.cn (Y.H.); an-peng@cau.edu.cn (P.A.); 2 Food Laboratory of Zhongyuan, Luohe 462300, China

**Keywords:** dandelion polysaccharides, atherosclerosis, blood lipid profiles, antioxidant, anti-inflammatory

## Abstract

Dandelion (*Taraxacum officinale*), a member of the Asteraceae (Compositae) family, is well known as the traditional medical plant. Dandelion polysaccharides, a natural active ingredient extracted from the dandelion, possess immune regulation, anti-inflammatory, antioxidant, and anti-aggregation properties. These properties suggest that dandelion polysaccharides might alleviate atherosclerosis. Using an *ApoE*^−/−^ atherosclerotic mice model fed a high-fat diet, we investigated the impact and potential mechanism of dandelion polysaccharides on atherosclerosis. We observed that dandelion polysaccharides significantly reduced the levels of triglyceride, total cholesterol, and low-density lipoprotein-cholesterol in serum, while elevated the high-density lipoprotein-cholesterol level. Concomitantly, dandelion polysaccharides reduced the area of atherosclerotic lesions and necrotic core of the aortic sinus, and increased the collagen content. Mechanistic studies showed that dandelion polysaccharides were effective in reducing serum malondialdehyde levels while elevating the enzymatic activities of superoxide dismutase and glutathione peroxidase. Furthermore, dandelion polysaccharides reduced the expression of chemotactic factor *Mcp-1* and pro-inflammatory cytokines (*Tnf-α*, *Il-1β*, and *Il-6*) in atherosclerotic lesions. Overall, these results indicated that dandelion polysaccharides may take an important part in the attenuation of atherosclerosis via its antioxidant and anti-inflammatory properties.

## 1. Introduction

Atherosclerosis (AS), characterized by atheroma or fibrous plaque formation through the accumulation of lipids and/or fibrous materials, is the leading cause of cardiovascular diseases (CVDs) [1,2]. According to the Heart Disease and Stroke Statistics 2020, deaths caused by CVD will reach 22.2 million by 2030 [3]. Excessive reactive oxygen species (ROS) and the inflammatory response have been widely observed in atherosclerosis [4,5]. However, the etiology and pathogenesis of atherosclerosis have not been completely elucidated. Blood lipid profiles, including triglyceride (TG), total cholesterol (TC), low-density lipoprotein (LDL), and high-density lipoprotein (HDL) have been considered as the causal risk factors for atherosclerotic CVDs [4,6]. Several drugs, including statins, ezetimibe, and inhibitors of the proprotein convertase subtilisin/kexin type (PCSK9), have been used in clinical practice to treat atherosclerosis [7]. However, long- term pharmacological therapy induced several safety concerns, such as liver damage, gallstones, myopathy, and acute renal failure [8,9]. Thus, more effectiveness and safer strategy for the treatment of atherosclerosis is needed.

Functional ingredients from natural herbs have shown their potentials in the treatment of metabolic diseases [10,11,12]. Dandelion (*Taraxacum officinale*), a nontoxic herb, has been considered as a valuable medical plant for its choleretic, diuretic, anti-rheumatic, and hepatoprotective qualities [13,14]. It contains various active ingredients, which included polysaccharides, flavonoids, peptides, terpenes, and a phenolic substance [15]. For example, dandelion leaf extract decreased serum lipids and insulin resistance in high-fat diet (HFD)-induced hepatic steatosis [16]. Dandelion root and leaf are also shown to display hypolipidemic and antioxidative effects to suppress the atherosclerosis on cholesterol-fed rabbits [17]. However, no specific ingredient was identified to exert the lipid-lowering effect so far. Dandelion flower water syrup decreased the vasoconstrictor prostanoids in the thoracic arteries of obese rats [13]. Studies have shown that dandelion polysaccharides perform well in immune regulatory, anti-inflammatory, and antioxidant properties. Dandelion polysaccharides showed better antioxidant effects [18]. The antimicrobial and antioxidant activities of dandelion polysaccharides prevented the metamorphism of fresh white shrimps [19]. Dandelion polysaccharides were shown to attenuate CCl_4_-induced liver injury through modulating inflammatory responses and ameliorating oxidative stress in rats [20]. In addition, immunomodulatory activities of dandelion extracts were capable to elevate multiple anti-inflammatory cytokines [21]. Furthermore, the root extract dandelion decreased lipid peroxidation in HepG2 cells [20,22,23].

The role of dandelion polysaccharides in the treatment of atherosclerosis remains unclear [24]. Because ROS and inflammation are two main factors triggering atherosclerosis, we hypothesized that dandelion polysaccharides may exert potential anti-atherosclerotic effects through antioxidant and anti-inflammatory properties. By measuring the pathological features of aorta roots, blood lipid profiles, antioxidant factors in serum samples, and inflammatory factors in aortas, we investigated the anti-atherosclerotic effect of dandelion polysaccharides in HFD-induced *ApoE*^−/−^ mice.

## 2. Materials and Methods

### 2.1. Animals

Six-week-old male *ApoE*^−/−^ mice were bought from Beijing Vital River Laboratory Animal Technology Limited Liability Company (Beijing, China). The mice were maintained under specific-pathogen-free conditions (temperature: 20–26 °C; humidity: 40–70%; pressure: 45 Pa; animal illumination: 15–20 Lux; light: 12 h/12 h light/dark cycle). To establish the animal model for atherosclerosis disease, all *ApoE*^−/−^ mice were fed with HFD diet, with 41% fat content and 4.7 kcal g^−1^ energy (H10141, Beijing HFK Bio-Technology Limited Liability Company, Beijing, China) for 10 weeks. Then, the *ApoE*^−/−^ mice were randomly assigned to a control group (saline) and experimental group (dandelion polysaccharides, 200 mg kg^−1^ d^−1^). Dandelion polysaccharides (≥90%, P1201031) were purchased from Beijing Kangruina Biotechnology Limited Liability Company (Beijing, China). The product was exacted from dandelion plant stems and roots by water extraction and alcohol precipitations. The purification procedures of dandelion polysaccharides were fractional precipitation, column chromatography, anion exchange chromatography, and gel chromatography. Dandelion polysaccharides powders were fully dissolved in the distilled water, and mixed without precipitation. According to the weight of each mouse, about a 200 μL volume of saline or dandelion polysaccharide solution was given in a daily oral gavage for 8 weeks. During the intervention period, weekly records were kept of both the body weight and the amount of feed consumed. All mice were sacrificed after anesthesia using 1.25% avertin intraperitoneally. Hearts and aorta tissues were harvested separately and stored in 4% formalin for histopathological study or −80 °C condition for biochemical experiments. All procedures of the study were strictly carried out according to the Guiding Principles for the Care and Use of Laboratory Animal, and were accepted by the Committee on the Ethics of Animal Experiments of China Agricultural University (Beijing, China).

### 2.2. Characterization of Dandelion Polysaccharides

Infrared spectroscopy of dandelion polysaccharides was analyzed by a Fourier transform infrared spectroscopy (FT−IR) spectrometer (Nicolet 6700, Thermo Scientific, Waltham, MA, USA) in the range of 4000–500 cm^−1^ at a resolution of 4 cm^−1^.

Molecular weight of dandelion polysaccharides was measured by gel permeation chromatography-high performance liquid chromatography (GPC-HPLC). The GPC-HPLC conditions were as follows: RID-20 refractive index indicator (Shimadzu, Kyoto, Japan); TSKgel GMPWXL column (Tosoh, Tokyo, Japan); Chromatographic mobile phase, 0.1 M NaNO_3_ and 0.05% NaN_3_ in pure water; flow rate, 0.6 mL/min; column temperature, 35 °C; injection volume, 20 μL.

Dandelion polysaccharides (20 μg) were made by hydrolysis-acidification by trifluoroacetic acid (2 M) at 120 °C for 4 h. Then, the derived dandelion polysaccharides and the standard monosaccharides (glucose, galactose, ribose, fucose, xylose, arabinose, rhamnose, mannose, glucuronic acid, and galacturonic acid) were analyzed on the LC-20AD HPLC (Shimadzu, Japan) using a Xtimate C18 column (4.6 × 200 mm, 5 μm). The HPLC conditions were as follows: Chromatographic mobile phase, 0.05 M potassium dihydrogen phosphate solution (PH 6.70); flow rate, 1.0 mL/min; column temperature, 30 °C; injection volume, 20 μL.

### 2.3. Analysis of Serum Lipid Profiles

After being left at room temperature for two to three hours, the collected blood samples of mice were centrifuged for 15 min at 3000 rpm. The serums were divided and kept until use at −80 °C. Levels of total TC, TG, LDL-C, and HDL-C in serum were measured according the commercially assay kits (S03027, S03042, S03029, S03025, Shenzhen Redu Life Sciences Limited Liability Company, Shenzhen, China).

### 2.4. Hematoxylin–Eosin (HE) Staining for Aorta

After being immersed and fixed for more than 48 h in the 4% paraformaldehyde, the mice aortas were embedded in paraffin for serial cross-sections (5 μm thick) using the microtome. Paraffin section samples prepared were stained by the commercial HE staining kit (G1120, Beijing Solarbio Science and Technology Limited Liability Company, Beijing, China). Serial slices stained were observed by optical microscope, and then analyzed for the atherosclerosis plaques by the Image J software (version 1.8.0, National Institutes of Health, Bethesda, MD, USA).

### 2.5. Oil Red O Staining for Lesions in Aorta

The aortic tissues were immersed in an optimal cutting temperature compound (OCT) at −80 °C. The prepared sections (10 μm thick) were sequentially stained with modified Oil Red O solution and counterstained with Mayer hematoxylin solution using the corresponding commercial assay kit (G1261, Beijing Solarbio Science and Technology Limited Liability Company, Beijing, China). The stained slices were observed for the pathological lesions under the Leica CTR6 microscope (Leica, Wetzlar, Germany).

### 2.6. Masson’s Trichrome Stain

Weigert’s iron hematoxylin solution, fuchsin ponceau acid solution, and aniline blue solution were used to stain the prepared paraffin sections according to the Masson’s trichrome stain kit instructions (G1340, Beijing Solarbio Science and Technology Limited Liability Company, Beijing, China). The optical microscope (Leica CTR6, Leica, Germany) was used to capture the Masson’s trichrome stain slices. The collagen proportion of stained slices were analyzed and quantified by Image J software (version 1.8.0).

### 2.7. Immunohistochemical Staining

The sections of samples were incubated with anti-Mac-3 antibody (1:100; 108501, Biolegend, San Diego, CA, USA) or anti-α-SMA antibody (1:100; 48938, Cell Signaling Technology, BSN, MA, USA) overnight at 4 °C. The sections were then incubated with the corresponding horseradish peroxidase-conjugated secondary antibody (HRP) (PV-9000, Beijing Zhong Shan-Golden Bridge Biological Technology Limited Liability Company, Beijing, China). The aortic slices were stained with 3,3′-diamino-benzidine tetrahydrochloride (DAB) (ZLI-9017, Beijing Zhong Shan -Golden Bridge Biological Technology Limited Liability Company, Beijing, China). The images were taken under an optical microscope, and Image J software (version 1.8.0) was used for the subsequent image analysis.

### 2.8. Analysis of MDA, SOD, and GSH-PX Levels in Serum

Malondialdehyde (MDA) levels in serum were assessed using the appropriate commercial assay kit (A003-1, Nanjing Jiancheng Technology Limited Liability Company, Nanjing, China). Superoxide dismutase (SOD) and glutathione peroxidase (GSH-PX) enzyme activities in serum were assessed using the corresponding commercial assay kit (A001-1, A005, Nanjing Jiancheng Technology Limited Liability Company, Nanjing, China).

### 2.9. Quantitative Real-Time PCR

Mice aortas were pulverized using an automatic freeze-grinding device at −20 °C. Total RNA was extracted by Trizol reagent (CW0580S, Cwbio, Beijing, China). NanoDrop 2000 microvolume spectrophotometers (Thermo Scientific, Waltham, MA, USA) were used to measure RNA concentration and purity. The following manufacturer’s instructions were strictly followed for the reverse transcription and real-time PCR procedures. With the guidance of HiScript III RT SuperMix (R323-01, Vazyme, Nanjing, China), RNA (1 μg) isolated from aorta samples was converted into cDNA. Real-time PCR was conducted using Taq Pro Universal SYBR qPCR Master Mix (Q712-02, Vazyme). The relative gene expression adopted the calculation method of 2^−ΔΔCt^. Table S1 details the primer sequences (*Actb*, *Tnf-α*, *Il-6*, *Il-1β*, and *Mcp-1*), which were synthesized by Beijing Genomics Institution (Beijing, China).

### 2.10. Immunoblotting Analysis

Protein samples from mice aortas were separated with 15% SDS-PAGE gel electrophoresis and then transferred to the PVDF membranes. The membranes were blocked with 5% skimmed milk for 2 h, and incubated with primary antibodies at 4 °C. After incubating with horseradish peroxidase-conjugated anti-mouse and anti-rabbit secondary antibodies, the body bindings were visualized using an ECL detection system (ChemiScope 6100, Shanghai Qinke Industrial Limited Liability Company, Shanghai, China) and analyzed by Image J software (version 1.8.0). The antibodies of inflammatory cytokines included *Tnf-α* (60291-1-1g, ProteinTech Group, Chicago, IL, USA), *Il-6* (ab259341, Abcam, Cambridge, UK), *Il-1β* (26048-1-AP, ProteinTech Group, Chicago, USA), and *Mcp-1* (ab308522, Abcam, Cambridge, UK).

### 2.11. Statistical Analysis

The results of experiments were presented as mean ± standard deviation (mean ± SD). GraphPad Prism statistical software (version 8, San Diego, CA, USA) was used to conduct the statistical analysis. *t*-test was used to evaluated statistical differences between the control group and experimental group. *p*-value < 0.05 represented the statistically significant.

## 3. Results

### 3.1. Characterization of Dandelion Polysaccharides

**Figure 1:**
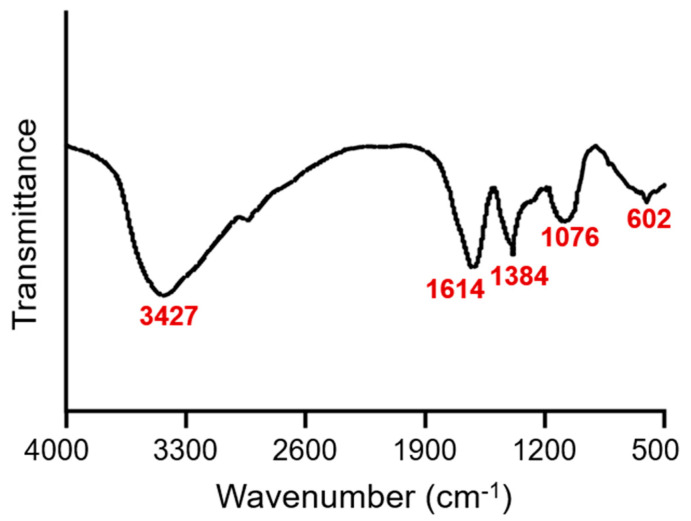
FT−IR spectrum of dandelion polysaccharides.

We first characterized the structure, molecular weight, and monosaccharide composition of dandelion polysaccharides in this study. In the FT−IR spectrum, the strong absorbance band at 3427 cm^−1^ was attributed to the stretching vibration of O–H (Figure 1). The bands at 1614 cm^−1^ and 1076 cm^−1^ indicated the stretching vibration of C–O. The band at 1384 cm^−1^ indicated the vibration of C–H. The weak band at 602 cm^−1^ was attributed to C–C stretching (Figure 1). The number average molecular weight (Mn), weight average molecular weight (Mw), and Z average molecular weight (Mz) of dandelion polysaccharides were 533, 4293, and 129,668, respectively (Table 1). The monosaccharide composition of dandelion polysaccharides was glucose, galactose, ribose, fucose, xylose, arabinose, rhamnose, mannose, glucuronic acid, and galacturonic acid in a molar ratio of 9.2:5.3:1.3:0.4:4.3:6.0:2.4:1.6:1.5:3.5 (Table 1).

### 3.2. The Establishement of Atherosclerotic Animal Model

**Figure 2:**
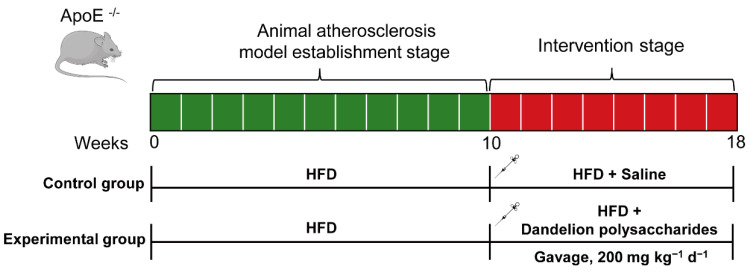
Scheme of dandelion polysaccharides intervention in HFD-induced atherosclerosis in *ApoE*^−/−^ mice.

**Figure 3:**
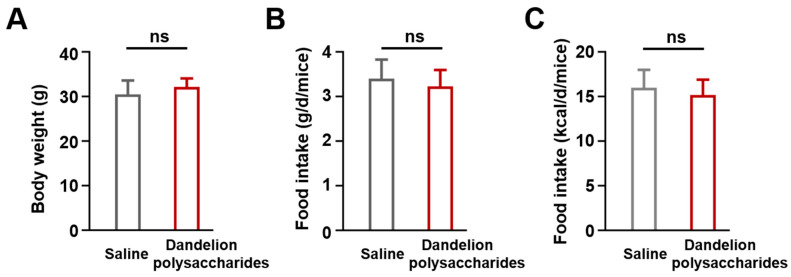
Effects of dandelion polysaccharides on body weight and food intake in HFD-induced *ApoE*^−/−^ mice. The body weight (**A**) and feed consumption (**B**,**C**) in dandelion polysaccharides and saline group. Results were presented as means ± SD, and n = 10 in each group. *t*-test, ns, *p* > 0.05.

In order to establish an atherosclerosis model, the *ApoE*^−/−^ mice were given an HFD for 10 weeks. Then, the atherosclerotic mice were randomly assigned to gavage saline or dandelion polysaccharides (200 mg kg^−1^ d^−1^, 200 μL) for an additional 8 weeks (Figure 2). We observed no difference between saline and dandelion polysaccharides groups in the final body weight (Figure 3A, *p* > 0.05). Moreover, both gram per day per mice and kcal per day per mice showed no difference in food intake between saline and dandelion polysaccharides groups (Figure 3B,C, *p* > 0.05). These results suggest that the treatment of *ApoE*^−/−^ mice with dandelion polysaccharides showed no effect on body weight or food intake.

### 3.3. Dandelion Polysaccharides Improved Lipid Profiles

**Figure 4:**
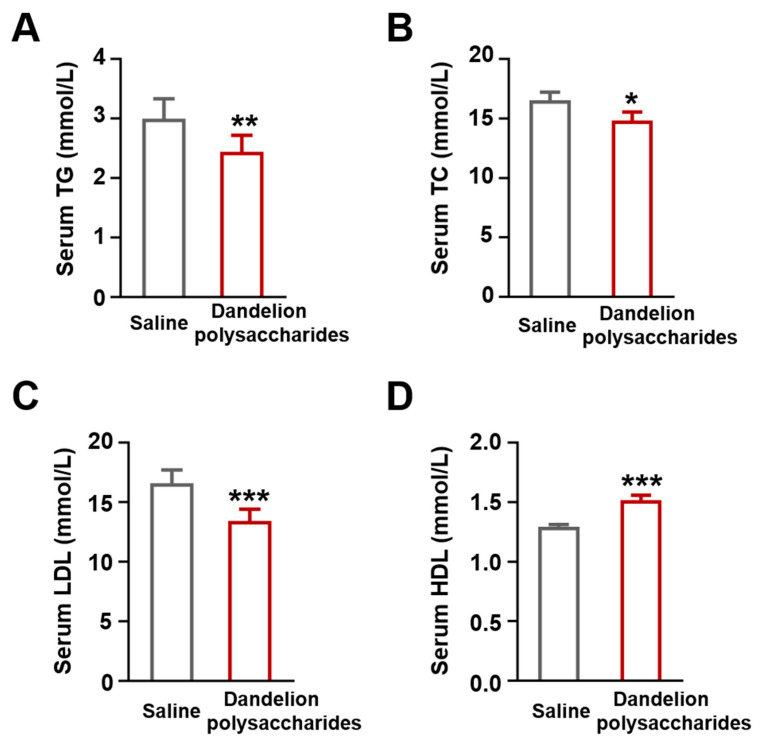
Effect of dandelion polysaccharides on serum lipids in HFD-induced *ApoE*^−/−^ mice. TG (**A**), TC (**B**), LDL-C (**C**), and HDL-C (**D**) levels of serum in dandelion polysaccharides and saline group. Results were presented as means ± SD, and n = 10 in each group. *t*-test, * *p* < 0.05, ** *p* < 0.01, *** *p* < 0.001.

Disordered lipid metabolism will promote atherosclerosis development [25]. Therefore, we next explored whether the administration of dandelion polysaccharides might influence the blood lipid profiles (TG, TC, LDL-C, HDL-C) in atherosclerotic mice. We found that dandelion polysaccharides reduced the levels of TG (*p* < 0.01), TC (*p* < 0.05), and LDL-C (*p* < 0.001), while increasing HDL-C level (*p* < 0.001) in serum samples (Figure 4A–D). These results indicated that dandelion polysaccharides intervention significantly impacted the serum lipid profiles in atherosclerotic mice.

### 3.4. Dandelion Polysaccharides Reduce Atherosclerotic Plaques in Aortic Roots

**Figure 5:**
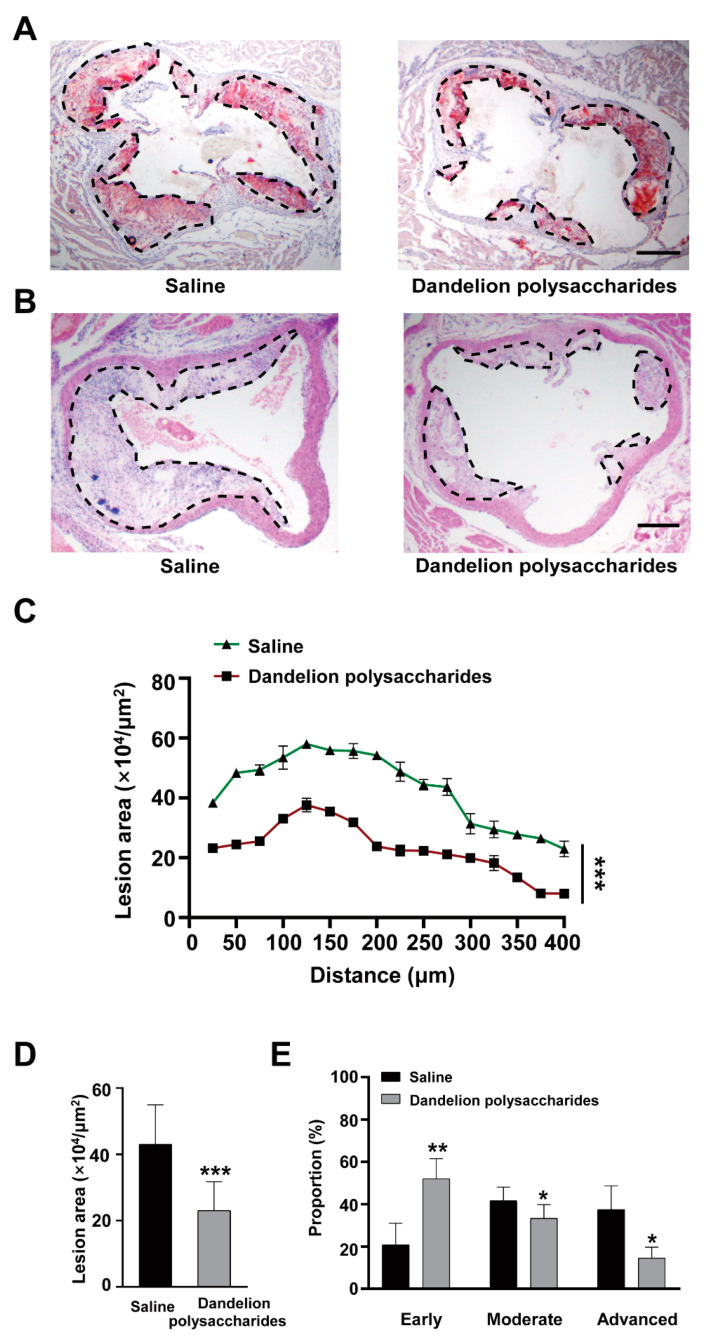
Effect of dandelion polysaccharides on atherosclerotic lesion in HFD-induced *ApoE*^−/−^ mice. Representative images for Oil red O staining (**A**), HE staining (**B**), quantitative chart (**C**), and curve chart (**D**) of atherosclerotic lesion area by HE staining. The proportion of early, moderate, and advanced atherosclerotic plaques based on histological staging (**E**). The sale bar is 250 μm. Results were presented as means ± SD and n = 10 in each group. *t*-test, * *p* < 0.05, ** *p* < 0.01, *** *p* < 0.001.

We next investigated whether the dandelion polysaccharides could reduce the plaque burden by examining the aortic roots in HFD-induced *ApoE*^−/−^ mice. The aortic sinus atherosclerotic lesion area was decreased in mice receiving oral gavage of dandelion polysaccharides (Figure 5A,B). Moreover, we observed that the lesion area of atherosclerotic plaques was reduced by 40% after dandelion polysaccharide treatment compared with the saline group (from 43 × 10^4^ μm^2^ to 23 × 10^4^ μm^2^) (Figure 5C,D).

To further explore whether dandelion polysaccharides could affect the progression of atherosclerosis, we classified the plaque into early, moderate, and advanced stages according to the Stary method [26]. The results suggested that dandelion polysaccharides-treated mice had a higher proportion of earlier plaques, and lower proportion of moderate and advanced plaques (Figure 5E). These results suggested that dandelion polysaccharides exerted an anti-atherosclerotic effect on HFD-induced *ApoE*^−/−^ mice.

### 3.5. Dandelion Polysaccharides Reduce Necrotic Core Area and Elevated Collagen Content of Plaques

**Figure 6:**
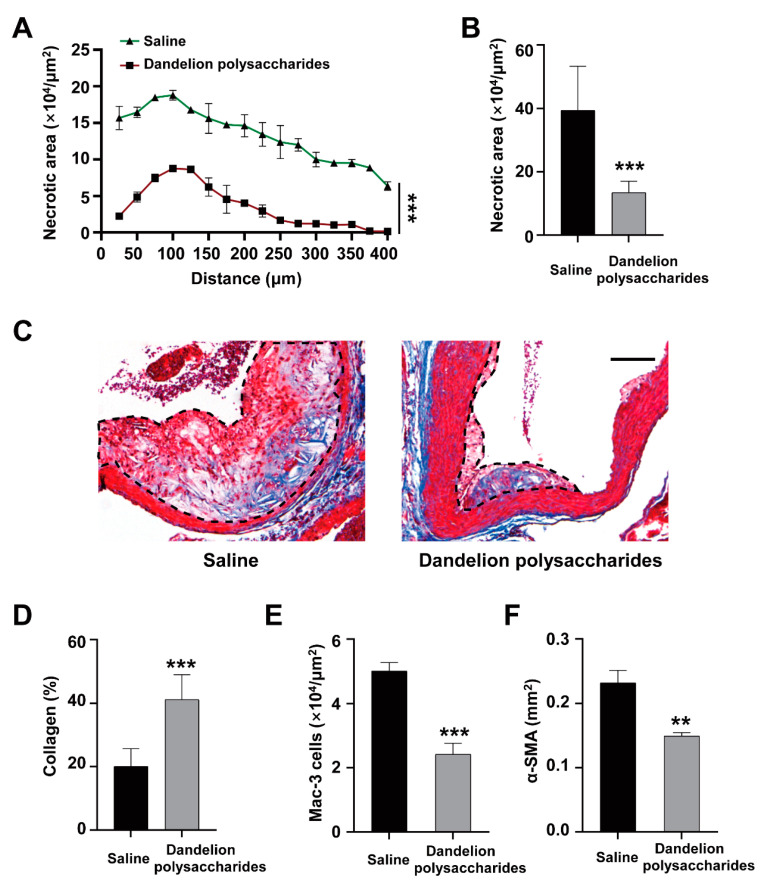
Effect of dandelion polysaccharides on plaques’ necrotic core and collagen in HFD-induced *ApoE*^−/−^ mice. Curve chart (**A**) and quantitative chart (**B**) of atherosclerotic necrotic core area. Representative images (**C**) and quantitative chart (**D**) of atherosclerotic collage area. Quantitative chart of macrophage number (**E**) and vascular smooth muscle cell area (**F**) in atherosclerotic plaque. The sale bar represents 150 μm. Results were presented as means ± SD, and n = 10 in each group. *t*-test, ** *p* < 0.01, *** *p* < 0.001.

Necrotic cores are responsible for inflammation, thrombosis, degradation of the proteolytic plaque, and physical stress on the fibrous cap, which are produced during the advanced lesion macrophages apoptosis and improper phagocytic clearance (or efferocytosis) of the apoptotic macrophages in advanced plaques [27]. We found that mice treated with dandelion polysaccharides had a smaller necrotic area (decreased by 60%, from 39 × 10^4^ μm^2^ to 13 × 10^4^ μm^2^) (Figure 6A,B, *p* < 0.001). In addition, dandelion polysaccharide administration also showed a higher percentage of collagen content (41%) than those in the saline group (20%) (Figure 6C,D, *p* < 0.001). These results suggested that dandelion polysaccharides might lead to more stable atherosclerotic lesions in atherosclerotic mice.

Macrophages and smooth muscle cells are major cells promoting artery remodeling and atherosclerosis [27,28]. To explore whether dandelion polysaccharides might impact the persistence of macrophages and smooth muscle cells, we next performed immunohistochemical staining of Mac-3 (marker for murine macrophage) and α-SMA (marker for smooth muscle cells). Compared to the saline groups, we found that the persistence of macrophages (Figure S1 and Figure 6E) and smooth muscle cells (Figure S2 and Figure 6F) in the atherosclerotic plaques were reduced in the dandelion polysaccharides group. The results indicated that dandelion polysaccharides inhibited the persistence of macrophages and smooth muscle cells within the atherosclerotic plaques.

### 3.6. Dandelion Polysaccharides Reduce the Levels of Oxidant-Related Markers in Serum Samples

**Figure 7:**
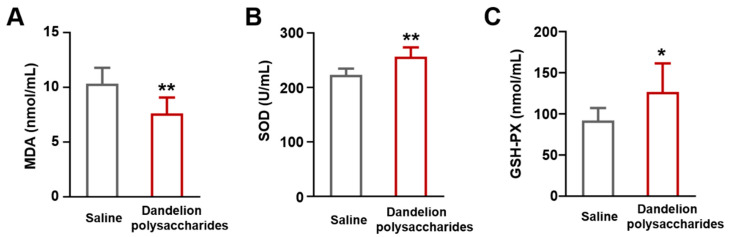
Effect of dandelion polysaccharides on antioxidant markers in HFD-induced *ApoE*^−/−^ mice. MDA concentration in serum (**A**). Activities of SOD (**B**) and GSH-PX (**C**) in serum. Results were presented as means ± SD, and n = 10 in each group. *t*-test, * *p* < 0.05, ** *p* < 0.01.

Previous studies demonstrated that polysaccharides possessed anti-oxidant capabilities [29]. The oxidative indicators included MDA, SOD, and GSH-PX [30]. MDA, a byproduct of lipid peroxidation, was a crucial signal for oxidative damage and lipid metabolism abnormalities during atherosclerosis development [31]. Therefore, we measured the levels of oxidative-related markers in serum samples from atherosclerotic mice. It was found that the dandelion polysaccharides group had lower MDA levels (Figure 7A, *p* < 0.01). Conversely, the levels of SOD and GSH-PX were elevated after the treatment of dandelion polysaccharides (Figure 7B, *p* < 0.01; Figure 7C, *p* < 0.05). These results suggest that dandelion polysaccharides inhibited atherosclerosis partially through its anti-oxidant capacity.

### 3.7. Dandelion Polysaccharides Reduce the Expression of Inflammatory Factors in Aorta

**Figure 8:**
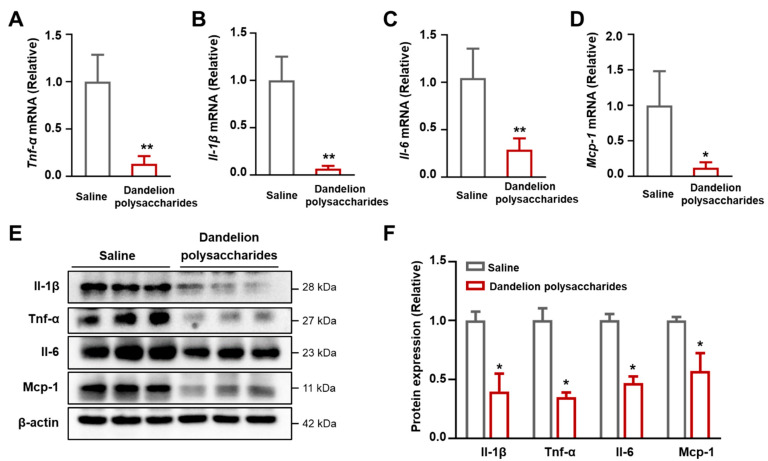
Effect of dandelion polysaccharides on chemotactic factors and inflammatory cytokines in HFD-induced *ApoE*^−/−^ mice. Relative mRNA expression of *Tnf-α* (**A**), *Il-1β* (**B**), *Il-6* (**C**), and *Mcp-1* (**D**) of mice aortas. (**E**) Immunoblotting of the expression of *Tnf-α*, *Il-1β*, *Il-6*, and *Mcp-1* in mice aortas from both saline and dandelion polysaccharides-treated groups. (**F**) Quantification of protein expression in E. Results were presented as means ± SD, and n = 10 in each group. *t*-test, * *p* < 0.05, ** *p* < 0.01.

Chemotactic factor (*Mcp-1*) and inflammatory cytokines (*Tnf-α*, *Il-1β*, and *Il-6*) are released during the initial stage of atherosclerosis, where they trigger the infiltration of monocytes across the endothelium and promote the formation of atherosclerotic plaques [32]. We therefore measured the mRNA and protein expressions of the chemotactic factor and inflammatory cytokines upon the administration of dandelion polysaccharides. The results suggested that the mRNA expression of inflammatory cytokines (*Tnf-α*, *Il-1β*, and *Il-6*) in the aortic tissues was greatly decreased in the dandelion polysaccharides group compared with the saline group (Figure 8A–C, *p* < 0.01). Concomitantly, the mRNA expression of the chemotactic factor (*Mcp-1*) was also decreased upon the intervention of dandelion polysaccharides (Figure 8D, *p* < 0.05). Meanwhile, the protein levels of these inflammatory cytokines and chemotactic factor in mice aortas were drastically decreased in the dandelion polysaccharides group (Figure 8E,F, *p* < 0.05). These results indicated that dandelion polysaccharides inhibited atherosclerosis partially through its anti-inflammatory response.

## 4. Discussion

Atherosclerosis is a chronic and progressive cardiovascular disease characterized by lipid dysfunction, oxidative stress, inflammation, migration of smooth muscle cells, formation of foam cells, covering of the fibrous cap, and accumulation of atheromatous plaques [33,34]. The phytochemicals derived from natural plants have been reported to improve atherosclerosis [35,36]. Dandelion polysaccharides were considered as one of the most important active compounds from the dandelion plant [37]. Emerging studies have proved that dandelion polysaccharides have a broad bioactive effect, such as anti-inflammatory and antioxidant activities [15,37]. These studies suggested that dandelion polysaccharides with multiple functions have a potential impact on the progression of atherosclerosis. To address this hypothesis, we explored the effect of dandelion polysaccharides on atherosclerosis in HFD-induced *ApoE*^−/−^ mice. In the atherosclerotic *ApoE*^−/−^ mice treated with saline or dandelion polysaccharides, we estimated the prospective impact of dandelion polysaccharides on the pathological characteristics of atherosclerotic lesions, lipid profiles, antioxidation markers in serum samples, and the expression of inflammatory cytokines in aortas.

In the present study, we used an *ApoE*^−/−^ atherosclerotic mice model to examine whether dandelion polysaccharides might exert anti-atherosclerotic effects. We showed that dandelion polysaccharides ameliorated high-fat-diet-induced atherosclerosis in mice through antioxidant and anti-inflammatory capabilities. The principal finding of this investigation is that dandelion polysaccharide intervention significantly inhibited the atherosclerotic plaques formation in aorta roots, which was considered as the most important site for the pathological assessment of atherosclerotic plaques [32]. Firstly, the area of atherosclerotic lesion upon the treatment of dandelion polysaccharides was reduced by 40% in polysaccharide-treated mice compared with controls. These results indicated that dandelion polysaccharides greatly reduced the atherosclerotic lesion area. Secondly, dandelion polysaccharides improved the blood lipid profiles in HFD-induced *ApoE*^−/−^ mice. Specifically, dandelion polysaccharides reduced the serum levels of multiple pro-atherosclerotic lipids, including TG, TC, and LDL-C. On the contrary, dandelion polysaccharides elevated atherosclerosis-protective HDL-C. These results are consistent with previous studies, showing that dandelion leave supplementation dramatically suppressed the levels of TG and TC [16,17]. Since lipid dysregulation is crucial in the development of atherosclerotic plaques [38], the reduction of atherosclerosis upon the treatment of dandelion polysaccharides might partially be attributed to its improving effect on blood lipid profile [17]. Thirdly, dandelion polysaccharides inhibited the serum oxidative stress in progressive atherosclerosis. Specifically, dandelion polysaccharides reduced the MDA level in serum samples and increased the SOD and GSH-PX levels. This observation was in line with earlier studies demonstrating that dandelion extract supplementation improved the lipid peroxidation [17,22]. The last finding of the present study is that dandelion polysaccharides reduced the expression of the chemotactic factor *Mcp-1* and pro-inflammatory cytokines. During the progression of atherosclerosis, the production of these inflammatory cytokines may promote endothelial damage [39]. In line with this study, the methanolic extract of dandelion reduced the pro-inflammatory cytokine expression in the LPS-induced endothelial cell [40].

Atherosclerosis, a chronic inflammation disease of the aorta, is a progress that involves the activation of abundant immune competent cells [41]. Proinflammatory cytokines and chemokines may trigger an unstable atherosclerotic plaque, subsequent atherothrombosis, and CVD events [42]. *Tnf-α*, a pro-inflammatory cytokine, promotes aortic inflammation during the progression of atherosclerosis [43]. *Il-1β* and *Il-6* have also been considered to play an important role in atherosclerotic plaque formation and development [44]. *Mcp-1*, a chemotactic factor for monocytes, was connected to the persistence of lipid-burden macrophages [45]. Hence, the elevated expression of inflammatory cytokines and chemokines may accelerate the rupture of the atherosclerotic plaque [46]. Immune modulatory therapies involving inflammation have been continually put forward to prove the possibility of anti-atherosclerosis [47,48,49]. However, the current treatments had undesirable adverse effects. Dandelion polysaccharides, as a natural safety ingredient, possess excellent anti-inflammation properties [18]. Therefore, we utilized this property of dandelion polysaccharides to treat atherosclerosis. As expected, we found reduced levels of *Tnf-α*, *Il-1β*, *Il-6,* and *Mcp-1* in aortic tissues upon treatment with dandelion polysaccharides. Consisted with this study, previous studies demonstrated that polysaccharides extracted from plants modulated inflammatory cytokines and chemokines [50,51,52]. Furthermore, studies also indicated that dandelion extracts exert anti-inflammatory effects. For instance, dandelion extracts had been found to reduce the LPS-induced expression of *Il-1β*, *Il-6*, and *Tnf-α* in rat skeletal muscle cells [53]. In addition, dandelion polysaccharides reduced the CCl_4_-induced liver damage via modulating NF-kB and its regulatory inflammatory mediators in rats [20]. This study also provided evidence that dandelion polysaccharides reduced the expression of chemotactic factors and inflammatory cytokines in atherosclerotic aorta.

Aside from the activation of pro-inflammation signaling pathways, as well as cytokine/chemokine expression, oxidative stress contributes to the pathogenesis of atherosclerosis [33]. Specifically, MDA is an important product of lipid peroxidation in atherosclerosis [54]. In contrast, the antioxidant systems SOD and GSH-PX have a reducing effect on lipid peroxidases and oxidized phospholipids. Since the pathophysiology of atherosclerosis is greatly influenced by oxidative stress [55], this study examined whether dandelion polysaccharides had an effect on oxidative stress in serum samples from atherosclerotic mice. The results showed that dandelion polysaccharide supplementation reduced the MDA level, while elevating the SOD and GSH-PX levels. This finding is in line with the observations that in atherosclerotic rabbits, dandelion root and leaf improved the activities of antioxidant enzymes in liver tissues [17]. Selenized polysaccharides from dandelion roots also showed antioxidant activities in vitro [14]. It has been reported that dandelion polysaccharide treatment prolonged the shelf life of white shrimps due to its antioxidant activities [18]. Similarly, two novel dandelion polysaccharides (DRP-2b, DRP-3a) had a good performance on the DPPH radical scavenging activity and hydroxyl radical scavenging ability [56]. Although these findings suggest that polysaccharides in dandelion may have anti-oxidant properties, they lack in vivo evidence. In this study, we provide direct evidence that dandelion polysaccharides showed antioxidant activities in HFD-induced *ApoE*^−/−^ mice. Therefore, the anti-atherosclerotic effect of dandelion polysaccharides may be attributable to its antioxidant capabilities.

**Figure 9:**
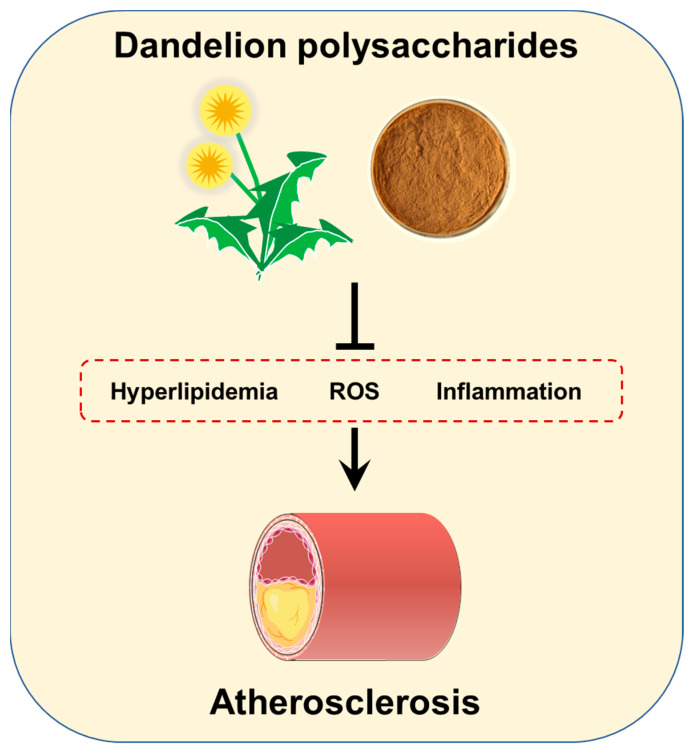
Schematic illustration of dandelion polysaccharides in ameliorating atherosclerosis. Compared with the saline group, the dandelion polysaccharide treatment improved the serum lipids and antioxidant markers, while lowering the expression of chemotactic factors and inflammatory cytokines in atherosclerotic mice.

In conclusion, this study identifies the anti-atherosclerotic activity of dandelion polysaccharides through its antioxidant and anti-inflammatory activities (Figure 9), and suggests that dandelion polysaccharides might serve as potential agents in the intervention of atherosclerosis.

## Figures and Tables

**Table 1:** Molecular weight and monosaccharide composition of dandelion polysaccharides.

Items	Amount
Number average molecular weight (Mn)	533
Weight average molecular weight (Mw)	4293
Z average molecular weight (Mz)	129,668
Glucose (μmol/L)	9.2
Galactose (μmol/L)	5.3
Ribose (μmol/L)	1.3
Fucose (μmol/L)	0.4
Xylose (μmol/L)	4.3
Arabinose (μmol/L)	6.0
Rhamnose (μmol/L)	2.4
Mannose (μmol/L)	1.6
Glucuronic acid (μmol/L)	1.5
Galacturonic acid (μmol/L)	3.5

## Data Availability

Not applicable.

## Supplementary Materials

The following supporting information can be downloaded at: https://www.mdpi.com/article/10.3390/nu15194120/s1, Table S1. Primers for Q-PCR analysis. Figure S1. Representative images of immunostained aortic plaques for macrophage using the macrophage marker Mac-3. The scale bar is 100 μm. Figure S2. Representative images of immunostained aortic plaques for smooth muscle cells using smooth muscle cells marker α-SMA. The scale bar is 100 μm.

## Abbreviation

ApoE^−/−^: apolipoprotein E knockout; AS: atherosclerosis; CVD: cardiovascular disease; FT−IR: Fourier transform infrared spectroscopy; GPC: gel permeation chromatography; HPLC: high-performance liquid chromatography; DAB: 3,3′-diamino-benzidine tetrahydrochloride; GSH-PX: glutathione peroxidase; HDL-C: high-density lipoprotein cholesterol; HE: hematoxylin–eosin; HepG2: human hepatocellular carcinoma cells; HFD: high-fat diet; IL-10: interleukin 10; IL-1β: interleukin 1 beta; IL-4: interleukin 4; IL-6: interleukin 6: LDL-C: low-density lipoprotein cholesterol; LDLr^−/−^: low density lipoprotein receptor knockout; Mac-3: macrophage-3; Mcp-1: monocyte chemoattractant protein 1; MDA: malondialdehyde; PBS: phosphate-buffered solution; ROS: reactive oxygen species; SD: standard deviation; SOD: superoxide dismutase; SPF: specific-pathogen-free; TC: total cholesterol; TG: triglyceride; Tnf-α: tumor necrosis factor a; α-SMA: α-smooth muscle actin.
