# Supplementary material for: Dandelion Polysaccharides Ameliorate High-Fat-Diet-Induced Atherosclerosis in Mice through Antioxidant and Anti-Inflammatory Capabilities
Source: Nutrients. 2023 Sep 24;15(19):4120. doi: 10.3390/nu15194120 (PMC10574455; doi:10.3390/nu15194120)
Supplement: Supplementary file 1 [file nutrients-15-04120-s001.zip › nutrients-2602872-supplementary.pdf]

Dandelion Polysaccharides Ameliorate High-Fat-Diet-Induced Atherosclerosis in Mice through Antioxidant and Anti-Inflammatory Capabilities

Shuaishuai Zhou [1], Zi Wang [1,2], Yanling Hao [1,2], Peng An [1,2], Junjie Luo [1,2,*] and Yongting Luo [1,2,*]

1 Department of Nutrition and Health, China Agricultural University, Beijing 100193, China; sszhou_2020@163.com (S.Z.); zxcvbnm7895123hh@163.com (Z.W.); haoyl@cau.edu.cn (Y.H.); an-peng@cau.edu.cn (P.A.)
2 Food Laboratory of Zhongyuan, Luohe 462300, China
* Correspondence: luojj@cau.edu.cn (J.L.); luo.yongting@cau.edu.cn (Y.L.)

Table S1. Primers for Q-PCR analysis.

Gene	Forward	Reverse
ACTB	GTGACGTTGACATCCGTAAAGA	GCCGGACTCATCGTACTCC
Tnf-α	ATGGCCTCCCTCTCATCAGT	TTTGCTACGACGTGGGCTAC
Il1β	TGCCACCTTTTGACAGTGATG	TGATGTGCTGCTGCGAGATT
Il6	CCCCAATTTCCAATGCTCTCC	CGCACTAGGTTTGCCGAGTA
Mcp-1	TTAAAAACCTGGATCGGAACCAA	GCATTAGCTTCAGATTTACGGGT

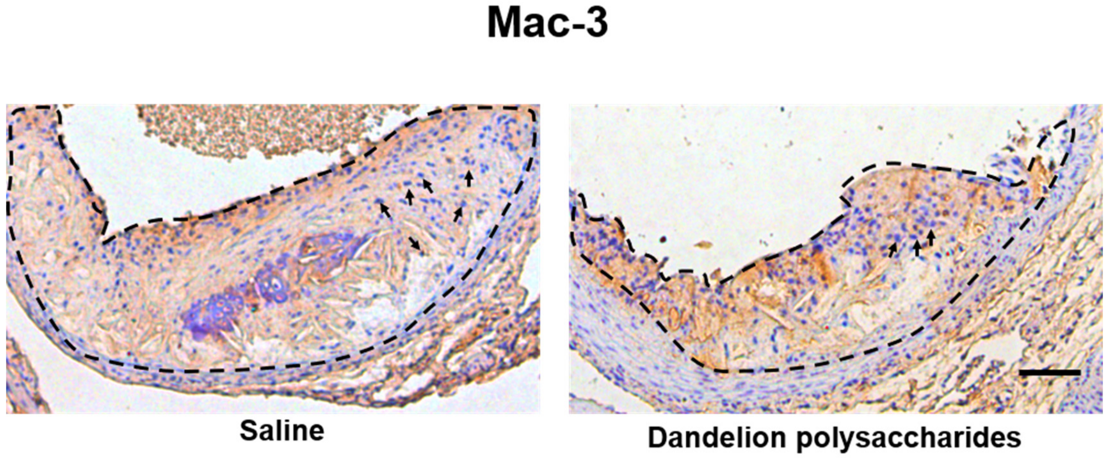

Figure S1. Representative images of immunostained aortic plaques for macrophage using the macrophage marker Mac-3. The scale bar is 100 μm.

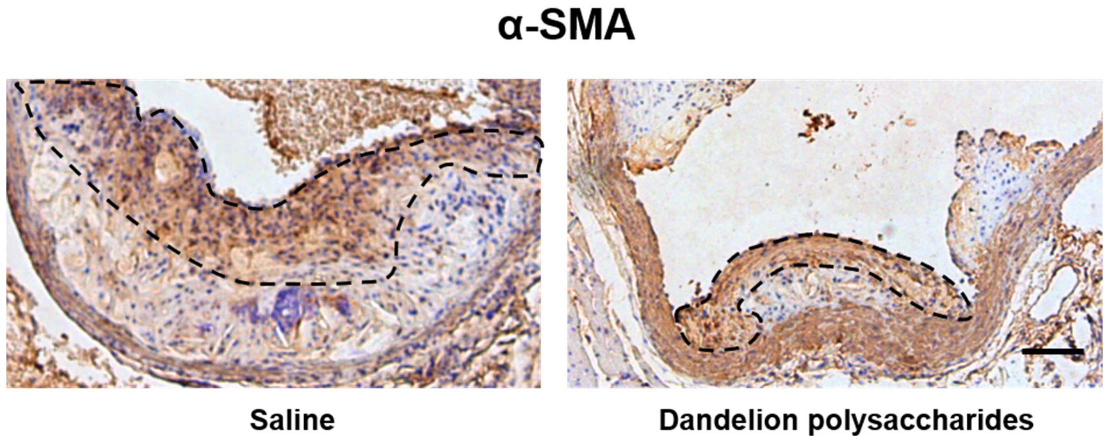

Figure S2. Representative images of immunostained aortic plaques for smooth muscle cells using smooth muscle cells marker α-SMA. The scale bar is 100 μm.
